# Elevated testicular apoptosis is associated with elevated sphingosine driven by gut microbiota in prediabetic sheep

**DOI:** 10.1186/s12915-022-01326-y

**Published:** 2022-05-24

**Authors:** Yuanchao Sun, Peng Sun, Yanting Hu, Liying Shan, Qi Geng, Yutian Gong, Haitao Fan, Teng Zhang, Yang Zhou

**Affiliations:** 1 State Key Laboratory of Reproductive Regulation & Breeding of Grassland Livestock, College of Life Sciences, Inner Mongolia University, Hohhot, 010070 China; 2 The Affiliated Hospital of Qingdao University and The Biomedical Sciences Institute of Qingdao University (Qingdao Branch of SJTU Bio-X Institutes), Qingdao University, Qingdao, 266003 China; 3 Laboratory of Microbiology and Immunology, College of Basic Medicine, Inner Mongolia Medical University, Hohhot, 010059 China

**Keywords:** Male infertility, Prediabetes, 16S-rDNA, Metabolome, Sphingosine, Melatonin, Fecal microbiota transplantation

## Abstract

**Background:**

Men with prediabetes often exhibit concomitant low-quality sperm production or even infertility, problems which urgently require improved therapeutic options. In this study, we have established a sheep model of diet-induced prediabetes that is associated with spermatogenic defects and have explored the possible underlying metabolic causes.

**Results:**

We compared male sheep fed a normal diet with those in which prediabetes was induced by a rich diet and with a third group in which the rich diet was supplemented by melatonin. Only the rich diet group had symptoms of prediabetes, and in these sheep, we found impaired spermatogenesis characterized by a block in the development of round spermatids and an increased quantity of testicular apoptotic cells. Comparing the gut microbiomes and intestinal digest metabolomes of the three groups revealed a distinctive difference in the taxonomic composition of the microbiota in prediabetic sheep, and an altered metabolome, whose most significant feature was altered sphingosine metabolism; elevated sphingosine was also found in blood and testes. Administration of melatonin alleviated the symptoms of prediabetes, including those of impaired spermatogenesis, while restoring a more normal microbiota and metabolic levels of sphingosine. Fecal microbiota transplantation from prediabetic sheep induced elevated sphingosine levels and impaired spermatogenesis in recipient mice, indicating a causal role of gut microbiota in these phenotypes.

**Conclusions:**

Our results point to a key role of sphingosine in the disruption of spermatogenesis in prediabetic sheep and suggest it could be a useful disease marker; furthermore, melatonin represents a potential prebiotic agent for the treatment of male infertility caused by prediabetes.

**Supplementary Information:**

The online version contains supplementary material available at 10.1186/s12915-022-01326-y.

## Background

Diabetes mellitus (DM) is a disorder of carbohydrate, fat, and protein metabolism caused by either insufficient insulin secretion or dysfunctional insulin use [1]. Currently, there are over 400 million adult patients with diabetes worldwide, with a prevalence in males of nearly 9.1%, while the trend is for rapid growth [2]. DM causes chronic and long-term damage to various organs, such as vision loss, decline in kidney function, and damage to the reproductive system [3]. Some patients of type 1 and type 2 DM suffer with testicular dysfunction and decreased spermatogenesis [4, 5]. Studies have shown that the expression of genes related to DNA repair in sperm nuclei is decreased in patients with type 1 DM. Meanwhile, the lack of sperm energy supply, due to mitochondrial DNA deletion, leads to a decrease in sperm motility [6]. In addition, it has been found that the increased levels of oxidative stress, reactive oxygen species (ROS), and lipid peroxidation in seminal plasma can cause reproductive dysfunction in the testes of patients with type 1 and type 2 DM [7–9]. Prediabetes is based on blood glucose parameters that are above normal but below the diabetes threshold and is a high-risk state for diabetes [10, 11]. Studies have shown that in addition to type 1 and type 2 DM, prediabetes also has a close connection with spermatogenesis [12, 13]. One report concerning prediabetic patients found that out of 744 infertile patients, 114 were undiagnosed prediabetic patients [13]. Another study also showed that serum total testosterone level decreased with age, while follicle-stimulating hormone (*FSH*) level and rate of non-obstructive azoospermia increased [12]. The above studies showed that in both DM and prediabetic patients, normal spermatogenesis was impeded due to abnormal metabolism.

In recent years, the regulation and improvement of gut microbiota to alleviate or treat diabetes has attracted clinical and research attention. In fact, numerous studies have already demonstrated the close relationship between the gut microbiota and DM [14–20]. Furthermore, reports have also shown that melatonin can regulate the composition of the intestinal flora and improve metabolic disorders caused by DM [21, 22]. Besides, the combined treatment (insulin plus melatonin) is reported to help Sertoli cells increase glucose consumption and produce more lactate, while the level of glycolysis-related enzymes and transporters is controlled in a suitable level within cultured Sertoli cells [23]. Another report also proved that adding 50 μM melatonin caused a higher level of lactate and alanine but a lower level of glycolysis-related transporters within cultured Sertoli cells, the increased level of lactates displayed an anti-apoptotic effect in male germ cells [24], indicating that melatonin may be an effective therapy in treatment of male infertility caused by DM. Moreover, recent studies have shown that changes in gut microbiota affect spermatogenesis [25–27]. Ding et al. report that the feeding of a high-fat diet induced metabolic disorders in mice resulting in failure of normal sperm development. They then transplanted the fecal microbes of metabolic disorder mice into normal mice, and the recipient mice began to suffer increasing levels of endotoxins, as well as testicular inflammation with damage to spermatogenesis [25]. Our previous study found that a reduction in the abundance of *Ruminococcaceae_NK4A214_group* influenced the absorption of vitamin A in a metabolic syndrome sheep model caused by the consumption of a high-energy diet, resulting in vitamin A deficiency in the testes and impaired spermatogenesis [27].

On this basis, we established a prediabetic sheep model and found that gut microbiota dysbiosis induced an increasing abundance of sphingosine, a metabolite of sphingomyelin. Sphingolipids are important components of cell membranes. Their metabolites, including ceramide (Cer), sphingosine (SPH), and sphingosine-1-phosphate (S1P) are important metabolic homeostatic devices for controlling the survival and apoptosis of cells and other life activities. Moreover, sphingosine is a negative regulator of cell proliferation, which plays a role in inhibiting cell growth and promoting cell apoptosis. From the general circulation, sphingosine accumulated in the testes, which led to an increased level of testicular cell apoptosis and an arrest in the transformation of S2 to S3 stage round spermatids in the prediabetes testis. We added melatonin to create a rescue group and found that it not only alleviated the symptoms of prediabetes but also significantly repaired sphingosine metabolism through actions of the gut microbiota. Furthermore, spermatogenesis and testicular cell apoptosis were also restored in the rescue group. Our prediabetic model revealed the effects of abnormal sphingosine metabolism caused by gut microbiota dysbiosis on spermatogenesis, and demonstrated the important role of melatonin in repairing this injury.

## Results

### Disordered spermatogenesis in a prediabetic model

**Fig. 1:**
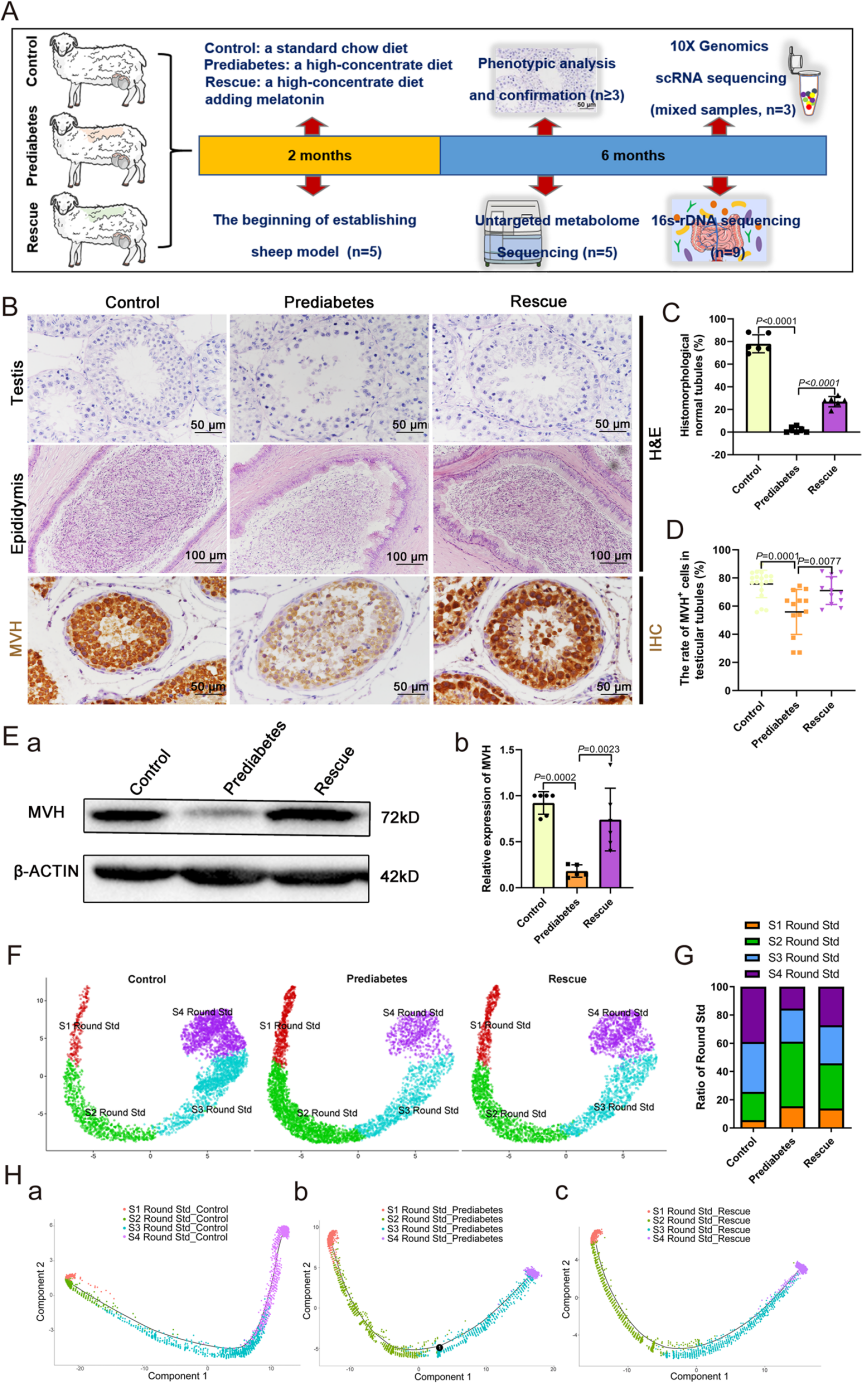
Disordered spermatogenesis in the prediabetic model. **A** The schematic to illustrate the experimental design. **B** Representative testicular and epididymal sections are stained with H&E and IHC for MVH. Scale bars = 100 μm and 50 μm. **C** The percentage of histomorphologically normal tubules in testicular samples from the three groups (*n* = 6). **D** The rate of MVH-positive cells in seminiferous tubules (*n* = 12). **E** (a) Western blot of MVH in the three groups. (b) Relative expression of MVH in each group (*n* = 6). **F** UMAP plot of round spermatids in testes, as defined by scRNA-seq analysis. **G** The percentage of four stages of round spermatids from the three groups. **H** (a-c) Single-cell trajectories of round spermatids in each group are shown with cells ordered in pseudotime. Branch point in the prediabetes group represents an arrest on transition from S2 to S3 Round Std

Several studies currently report the close relationship between prediabetes and spermatogenesis [12, 13, 28, 29], but the underlying mechanism is unclear. Hence, we induced a sheep model of prediabetes through feeding a high-concentrate diet and subsequently added melatonin as the rescue group (Fig. 1A). In the prediabetic model, body weight, fasting insulin, fasting glucose levels, and the HOMA-IR index increased significantly compared with those of the control group, while decreasing trends were found in the rescue group (Additional file 1: Fig. S1A-B). Serum lipoprotein is one type of lipid-protein complex, for example high-density lipoprotein (HDL), which mitigates levels of atherosclerotic lipoprotein, as well as low-density lipoprotein (LDL); the latter transports cholesterol, which can be deposited within arterial walls causing cardio-cerebral vascular diseases. HDL levels were decreased, while LDL levels were significantly increased in the prediabetic group (Additional file 1: Fig. S1C). In addition, compared with the control group, the serum total cholesterol (CHOL2) and hepatic lipase (LIPC) of the prediabetic sheep model were significantly increased, while the rescue group showed significant protective effects (Additional file 1: Fig. S1C). The above data indicated that we successfully created a sheep model of prediabetes.

In humans, some people with undiagnosed prediabetes suffer infertility [13]. We initially examined the morphology of the ovine testes and epididymides and found that there was a phenomenon of spermatogenesis block in prediabetic sheep testes. Specifically, abnormal seminiferous tubules were found, and significant cell debris was observed within the epididymides in the prediabetes group. We also observed that the conjunction between spermatogenic cells and Sertoli cells declined; some of the spermatogenic cells even separated from Sertoli cells and gathered in the center of the seminiferous tubule (Fig. 1B), resulting in a decreased percentage of normal seminiferous tubules (Fig. 1C). At the same time, protein expression levels of mouse vasa homolog (MVH) and MVH positive cells were significantly lower than in the control group, exhibiting significant spermatogenic cell damage in the prediabetes group (Fig. 1D, E). Compared with the prediabetes group, the quality and quantity of sperm in seminiferous tubules and epididymides of testes were significantly increased in the rescue group, and the number of MVH positive cells was also increased, displaying a noticeable improvement in sperm development compared to the prediabetes group (Fig. 1B–E, Additional file 1: Fig. S2A-B).

To explore the gene expression profile in testicular tissue from the three groups, we performed single-cell transcriptome sequencing analysis using the 10X Genomics platform. In this study, spermatogenic cells were mainly divided into six clusters, spermatogonia cell (SPG), early spermatocyte cells (early SPC), late SPC, early round spermatid (Early Round Std), Mid Round Std, and Late Round Std. Somatic cells were mainly identified as two types: Sertoli cells and Leydig cells (Additional file 1: Fig. S3A-B). We then analyzed the differences of gene expression among different stages of spermatogenic cells, while the expression distribution of spermatogenic cells was analyzed using pseudotime sequencing. When performing pseudotime distribution and cell type analysis in SPG, SPC, and Round Std of the three experimental groups, we found that there was a distinct developmental arrest in Round Std of the prediabetes group. Initially, we divided Round Std into four stages, S1-S4 Round Std, and performed statistical analysis to count their ratio in each group (Fig. 1F). Distinct differences were revealed in S2 Round Std (control: 20%, prediabetes: 45.7%, rescue: 32%) and S3 Round Std (control: 35.4%, prediabetes: 23.5%, rescue: 27%; Fig. 1G), indicating there was an S2 Round Std arrest in the prediabetes group. Subsequently, we performed pseudotime distribution analysis of S1 to S4 Round Std in each group; we found that there was a branching phenomenon from S2 to S3 Round Std, indicating an arrest in S2 Round Std in the prediabetes group (Fig. 1H), which coincided with the statistical analysis of the Round Std ratio. In addition, we performed gene expression profile analysis on the transition point of S2 Round Std arrest and found that the differential genes were enriched in sperm flagella assembly and sperm motility (Additional file 1: Fig. S3C-D).

### Abnormal metabolism of sphingosine in the prediabetic model

**Fig. 2:**
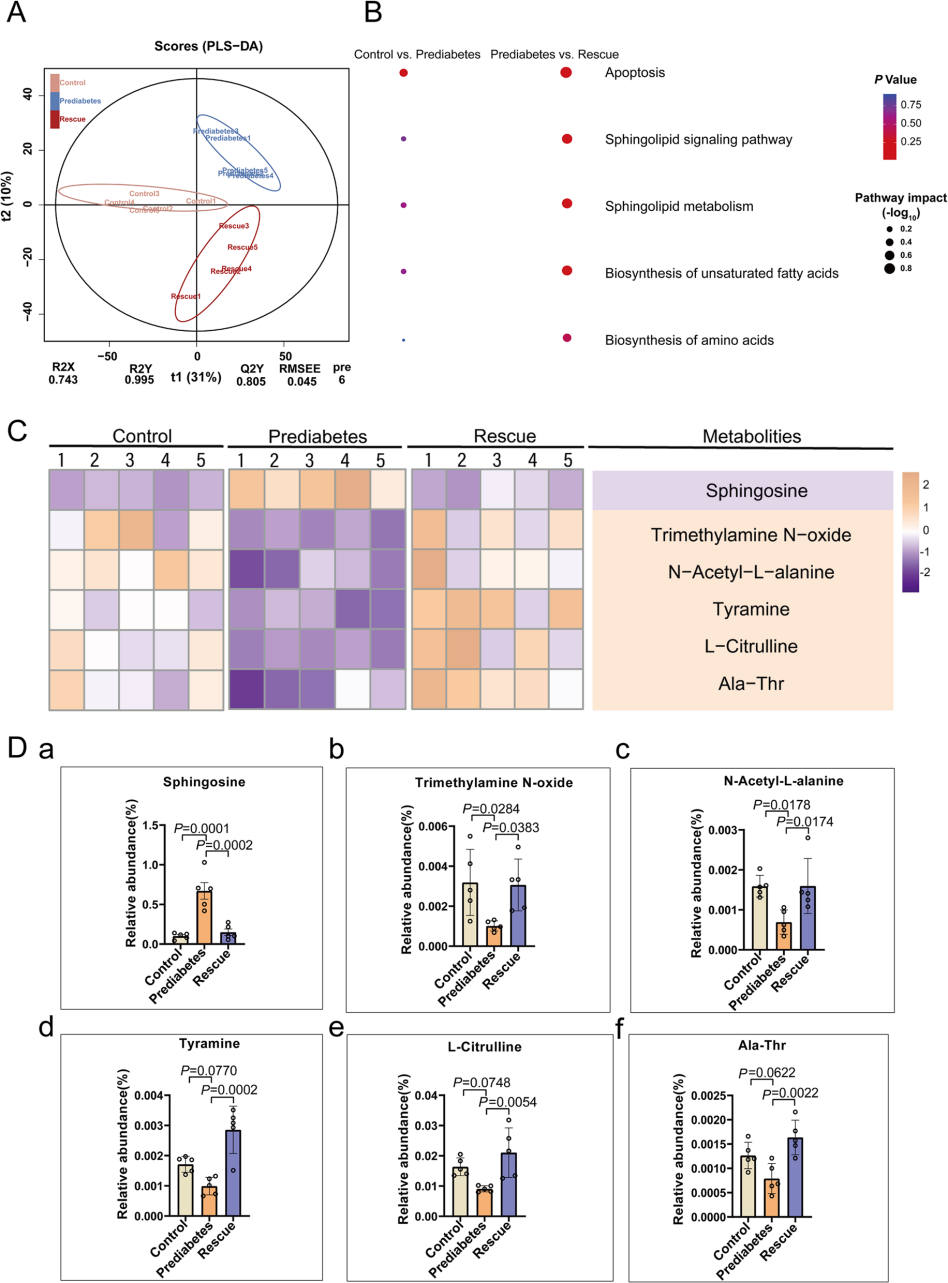
Abnormal metabolism of sphingosine in the prediabetic model. **A** PLS-DA score plot for discriminating the intestine digesta metabolome from the three groups. **B** Disturbed metabolic pathways in the control vs. prediabetes, and prediabetes vs. rescue groups. **C** Heatmaps of the differential metabolites that were altered in the prediabetes and rescue groups compared with the control group. **D** Comparison of the relative abundance of (a) sphingosine, (b) trimethylamine N-oxide, (c) N-acetyl-L-alanine, (d) tyramine, (e) L-citrulline, and (f) Ala-Thr in the three groups (*n* = 5)

Mounting studies have shown that an imbalance of substance metabolism in diabetic patients affects their spermatogenesis [4, 5, 25–27]. Therefore, we examined the metabolomic characteristics of the three groups to explore the association between testicular development and prediabetic substance metabolism. Here, we used UHPLC/Q-TOF MS (ultra-high performance liquid chromatography-quadrupole time-of-flight mass spectrometry) to assess the metabolome profiles of intestinal digesta (Additional file 1: Fig. S4A-C). Firstly, we performed PLS-DA (partial least squares discriminant analysis) clustering of the metabolites of the three groups and found a significant difference between the control and prediabetes group, while the rescue group resembled the control (Fig. 2A), displaying a protective effect. Then, we analyzed the difference in differential metabolites and found they were mainly enriched during apoptosis and in the sphingolipid metabolism pathway (Fig. 2B). We isolated the top six differential metabolites through heatmap analysis, as shown in Fig. 2C, D and Additional file 1: Fig. S4D-F, and found that sphingosine was remarkably increased in prediabetes, its difference was significantly greater than the other five. Sphingosine is a metabolite of sphingomyelin, which plays a vital role in promoting cell apoptosis. Next, we aimed to explore the influence of sphingosine accumulation on spermatogenesis.

### Sphingosine accumulation correlates with impaired spermatogenesis in the testicles of the prediabetic sheep

**Fig. 3:**
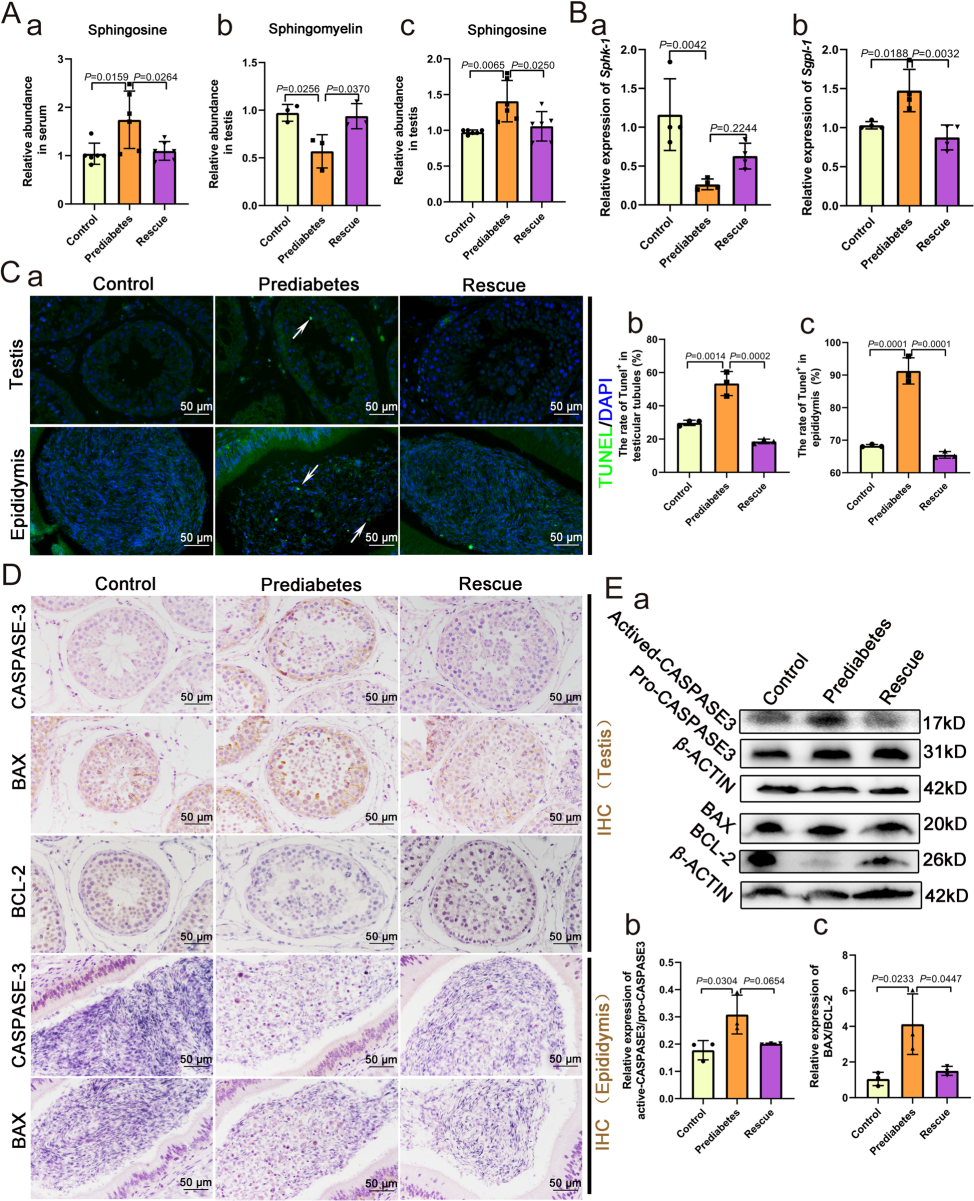
Sphingosine accumulation affects spermatogenesis in the testes of the prediabetic model. **A** The relative abundance of (a) sphingosine in serum of the three groups (*n* = 6). The relative abundance of (b) sphingomyelin (*n* = 3) and (c) sphingosine in testes of the three groups (*n* = 6). **B** The relative expression of (a) *Sphk-1* (*n* = 4) and (b) *Sgpl*-1 (*n* = 4) in testicular tissues of the three groups. **C** (a) TUNEL staining of representative sections within testicular and epididymal tissues of the three groups, and the rate of TUNEL-positive cells in (b) testicular tubules (*n* = 3) and (c) epididymides (*n* = 3). **D** IHC staining for CASPASE-3, BAX, and BCL-2 in representative sections of testicular and epididymal tissue of the three groups. **E** (a) Western blot of Actived-CASPASE-3, Pro-CASPASE-3, BCL-2, BAX, and β-ACTIN in the three groups; the relative expression of (b) Actived-CASPASE-3/Pro-CASPASE-3 (*n* = 3) and (c) BAX/BCL-2 (*n* = 3) in the three groups

To confirm the effects of the abnormal metabolism of sphingosine on spermatogenesis, we detected the levels of sphingomyelin and sphingosine in the three groups and found that serum levels of sphingosine were increased in prediabetic sheep. In the testes of prediabetic sheep, sphingomyelin levels were decreased while the abundance of sphingosine increased (Fig. 3A), indicating that sphingosine accumulation takes place in the testes from the general circulation. In addition, the expression levels of *Sphk-1* and *Sgpl-1* related to sphingosine metabolism were significantly altered in prediabetic sheep testes (Fig. 3B). In order to study the influence of increased sphingosine abundance on spermatogenesis in testes, TUNEL staining of testicular and epididymal tissues was performed in the three groups. In the prediabetic model, TUNEL positive cell numbers were increased in tissues of both the testis and epididymis (Fig. 3C). In the rescue group, the addition of melatonin had a mitigating effect. Through immunohistochemical staining, we found that the expression of apoptotic path-related BAX protein and CASPASE-3 protein were increased in prediabetes testes and epididymis, whereas the expression of BCL-2 was decreased in prediabetic testes (Fig. 3D). Western blot results also confirmed these findings, the relative expression of both actived-CASPASE3 and BAX/BCL-2 significantly increased, showing that there was an obvious increased level of apoptosis in prediabetic testes (Fig. 3E). The rescue group showed a protective effect. In fact, we found that in addition to spermatogenic cells, Sertoli cells also exhibited an increased apoptotic signal in prediabetic sheep testes. Thus, we suspected that the accumulation of sphingosine might have an impact on the status of Sertoli cells. Therefore, we analyzed the transcriptome level of Sertoli cells in the three groups (Additional file 1: Fig. S5A-B). It was found that the expression of spermatogenesis and cilium assembly related genes decreased, while the expression of exocytosis and cellular component transport related genes increased in the prediabetes group compared to the control group (Additional file 1: Fig. S5C). We also demonstrated that the gene *Psap*, which encodes a multifunctional glycoprotein that acts in the intracellular metabolism of sphingolipids, and the gene *Bcap31*, whose encoded protein is involved in CASPASE 8-mediated apoptosis, were significantly upregulated in the prediabetes group (Additional file 1: Fig. S5D), predicting that Sertoli cells were also influenced by the accumulation of sphingosine. Considering that Sertoli cells play essential roles in supporting and feeding spermatogenic cells, we suggest that sphingosine accumulation affects the status of Sertoli cells, which decreases the conjunction between Sertoli cells and spermatogenic cells, and in turn causes an increased level of apoptosis in spermatogenetic cells.

### Prediabetic model suffers with gut microbiota dysbiosis

**Fig. 4:**
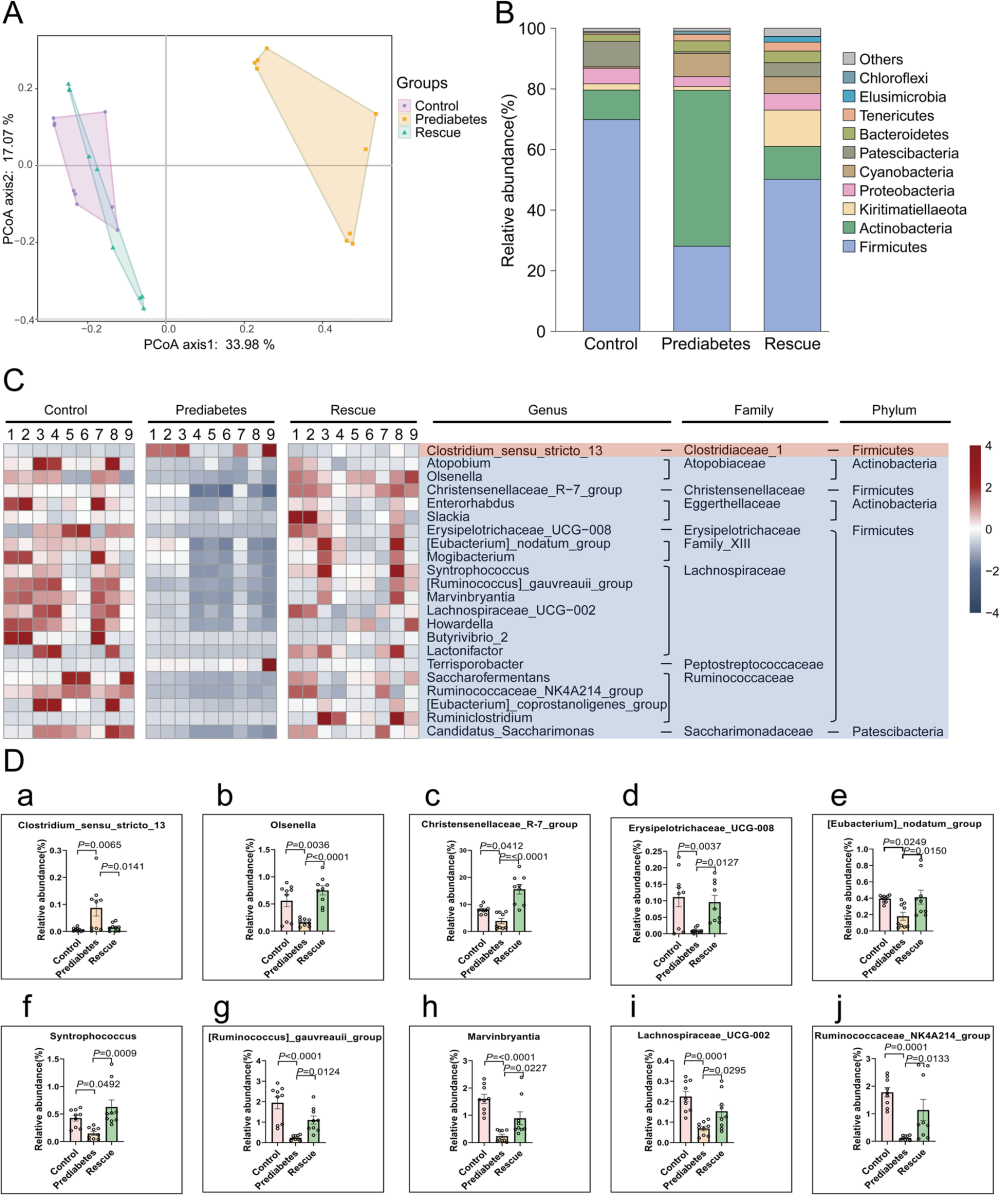
Prediabetic sheep suffer gut microbiota dysbiosis. **A** PCoA plot of gut microbiota based on the operational taxonomic unit metrics of samples in the three groups. **B** Bacterial taxonomic profiling at the phylum level of intestinal bacteria from the three groups. **C** Heatmap of differential abundance from the prediabetes and rescue groups, compared with the control group. **D** The differential abundance of intestinal bacteria at the genus level of (a) *Clostridium_sensu_stricto_13*, (b) *Olsenella*, (c) *Christensenellaseae_R-7_group*, (d) *Erysipelotrichaceae_UCG_008*, (e) *[Eubacterium]_nodatum_group*, (f) *Syntrophococcus*, (g) *[Ruminococcu]_gauvreauii_group*, (h) *Marvinbyrantia*, (i) *Lachnospiraceae_UCG_002*, and (j) *Ruminococcaceae_NK4A214_group* from the prediabetes and rescue groups compared with the control group (*n* = 9)

**Fig. 5:**
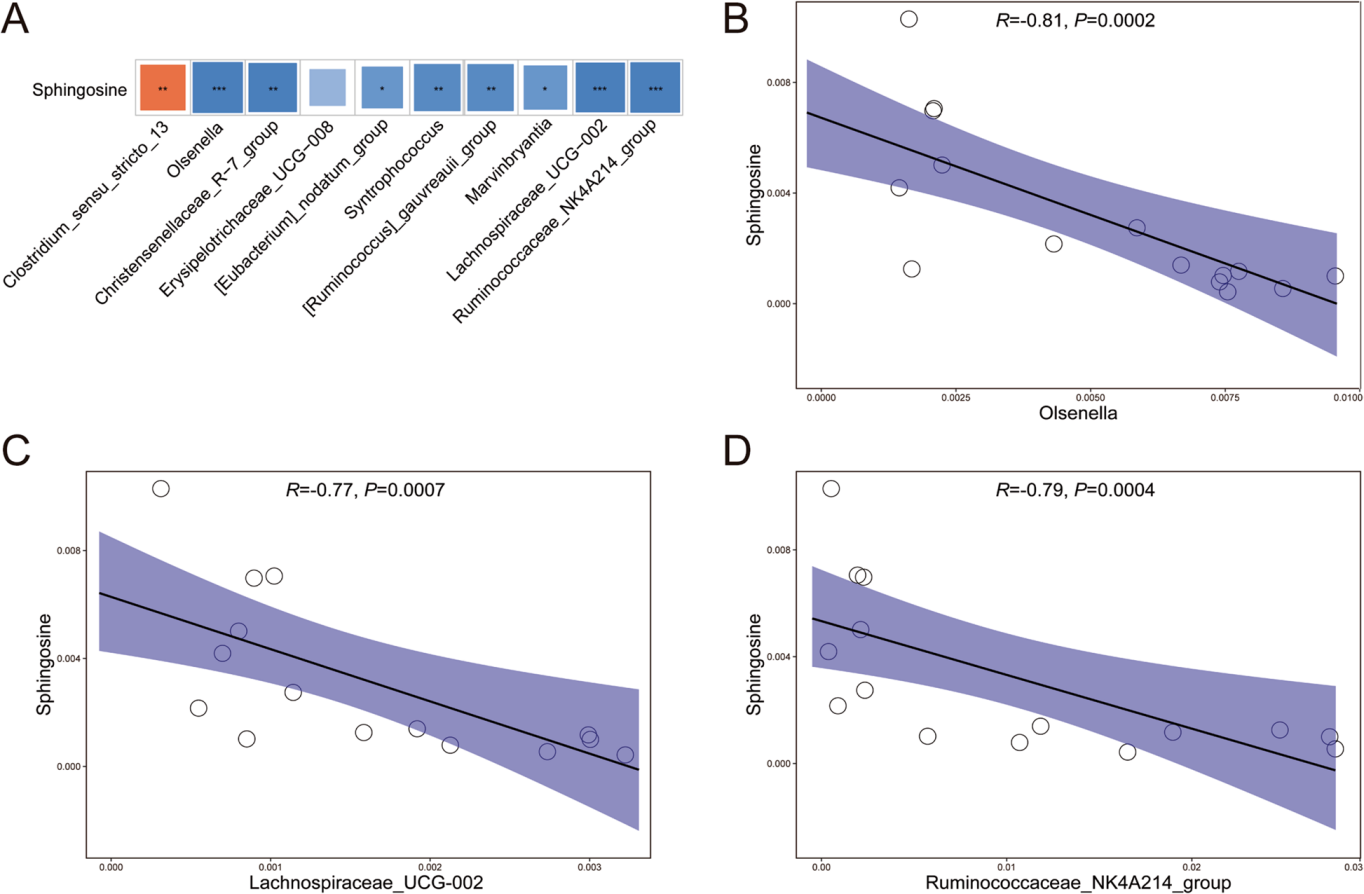
The functional relationship between differential microbiota at the genus level with sphingosine. **A** Spearman’s correlation analysis of rescued differential microbiota at the genus level with sphingosine. **B** Spearman’s correlation analysis of *Olsenella* abundance with sphingosine. **C** Spearman’s correlation analysis of *Lachnospiraceae_UCG_002* abundance with sphingosine. **D** Spearman’s correlation analysis of *Ruminococcaceae_NK4A214_group* abundance with sphingosine

The type and composition of gut microbiota play an important role in metabolic activity within the host [30]. Several studies have already confirmed that the gut microbiota is closely related to diabetes and spermatogenesis [17, 18, 22, 25]. Therefore, we examined changes in the composition and proportion of gut microbiota by performing 16S rDNA sequencing of intestinal digesta, to examine gut microbiota profiles in the three groups. Through Bray–Curtis principal coordinate analysis (PCoA), we found that the gut microbiota profiles in the prediabetes group were significantly different from the control group, whereas the rescue group was quite similar to the control group (Fig. 4A). We also analyzed the degree of bacterial taxonomic similarity in the three groups to assess the composition of the bacterial community at the phylum level. Among them, *Firmicutes*, which has a large proportion of bacterial community members, was significantly decreased in the prediabetes group, while *Actinobacteria* was significantly increased (Fig. 4B). The rescue group showed an obvious protective effect, which was similar to the control group. Furthermore, biological abundance and diversity index of gut microbiota, using the Shannon index, was significantly decreased in the prediabetes group compared to the other two groups (Additional file 1: Fig. S6A). At the genus level, we found that 22 bacteria were altered in the three groups (Fig. 4C. and Additional file 1: Fig. S6B); as shown in Fig. 4D, there were 10 bacteria that showed rescue effects among the three groups. By using Spearman’s correlation analysis, we investigated the functional relationship between differential genus microbiota levels with sphingosine (Fig. 5A). Sphingosine showed a significant negative correlation with *Olsenella*, *Lachnospiraceae_UCG_002*, and *Ruminococcaceae_NK4A214_group* (Fig. 5B–D)*.* The above data revealed that in prediabetic sheep, there was an obvious change in the constitution of gut microbiota compared to the control group. Among them, *Olsenella*, *Lachnospiraceae_UCG_002*, and *Ruminococcaceae_NK4A214_group* showed a significant difference and a close relationship with sphingosine metabolism, indicating that abnormal metabolism of sphingosine in the prediabetic group was associated with changes in the gut microbiota.

### Fecal microbiota transplantation confirmed the influence of microbiota dysbiosis on spermatogenesis

**Fig. 6:**
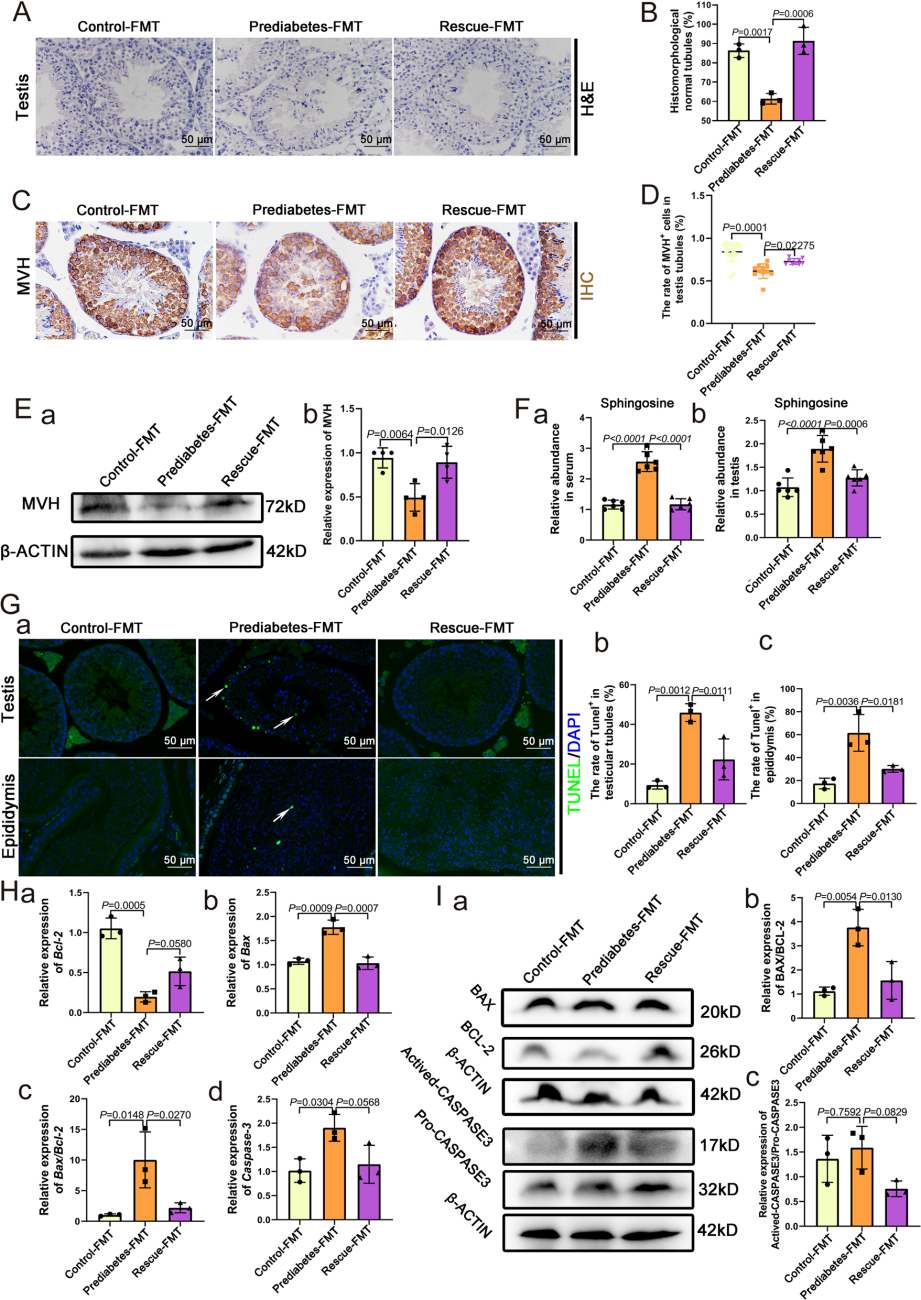
Fecal microbiota transplantation to confirm the influence of microbiota dysbiosis on spermatogenesis. **A** H&E staining of control-FMT, prediabetes-FMT, and rescue-FMT testicular tissue. **B** Percentage of normal seminiferous tubules in testes of the three groups (*n* = 3). **C** IHC staining for MVH in representative sections of the testicular tissues of the three groups. **D** The rate of MVH-positive cells within seminiferous tubules in testes of the three groups (*n* = 9). **E** (a) Western blot of MVH in the three FMT groups. (b) Relative expression of MVH in each group (*n* = 4). **F** The relative abundance of (a) sphingosine in serum and (b) testes of the three FMT groups (*n* = 6). **G** (a) TUNEL staining of representative sections within testicular and epididymal tissues of three FMT groups, and the rate of TUNEL-positive cells in (b) testicular tubules (*n* = 3) and (c) epididymides (*n* = 3). **H** The relative expression of (a) *Bcl-2*, (b) *Bax*, (c) *Bax*/*Bcl-2*, and (d) *Caspase3* in testes of the three FMT groups (*n* = 3). **I** (a) Western blot of Actived-CASPASE-3, Pro-CASPASE-3, BCL-2, BAX, and β-ACTIN in the three FMT groups; the relative expression of (b) BAX/BCL-2 and (c) Actived-CASPASE-3/Pro-CASPASE-3 in the three FMT groups (*n* = 3)

The results in the prediabetic model indicated that gut microbiota dysbiosis contributed to sphingosine accumulation and then increased apoptosis in testicular tissues. To confirm this, fecal microbiota transplantation (FMT) was performed in acceptor mice. In FMT mice, there was no obvious difference in body weight and blood glucose tolerance test among the three groups (Additional file 1: Fig. S7A-C). To confirm the efficiency of FMT, we detected the abundance of *Olsenella* in the FMT mice by PCR and qPCR and found that the abundance of *Olsenella* was significantly decreased in the prediabetes-FMT group compared with control-FMT group while it was increased in the rescue-FMT group compared to the prediabetes-FMT group (Additional file 1: Fig. S8A-B). The status of spermatogenesis exhibited significant differences. As shown in Fig. 6A, B, the prediabetes-FMT group showed abnormal spermatogenesis, and some of the spermatogenic cells were even detached from the Sertoli cells, and the ratio of histomorphologically normal tubules significantly decreased; meanwhile, the rescue-FMT group showed a protective trend compared to the prediabetes group. Following MVH staining, we found the rate of MVH-positive cells within the seminiferous tubules was significantly reduced in the prediabetes-FMT group (Fig. 6C, D), and this was confirmed by western blot, where the expression of MVH was significantly decreased (Fig. 6E). To confirm that gut microbiota dysbiosis contributed to sphingosine accumulation, we detected the levels of sphingosine in the three FMT groups and found that serum and testicular levels of sphingosine were increased in prediabetes-FMT group, and these levels of sphingosine were decreased in the rescue-FMT group, compared to the prediabetes-FMT group (Fig. 6F). With TUNEL staining, we found an increased level of apoptosis in the prediabetes-FMT group, and a higher rate of TUNEL positive cells in the epididymal tissues, compared to the control-FMT group, while the rescue-FMT group showed a protective effect (Fig. 6G). Through real-time quantitative PCR analysis, we found that the mRNA level of *Bax* and *Caspase-3* were increased, whereas the level of *Bcl-2* was decreased in prediabetes-FMT group (Fig. 6H). Western blot results also confirmed that the relative protein level of both actived-CASPASE3/pro-CASPASE3 and BAX/BCL-2 increased in prediabetes-FMT group, while the rescue-FMT group showed a protective effect (Fig. 6I). The FMT results confirmed that gut microbiota dysbiosis induced sphingosine accumulation, which led to increased apoptosis level in testis and epididymis.

**Fig. 7:**
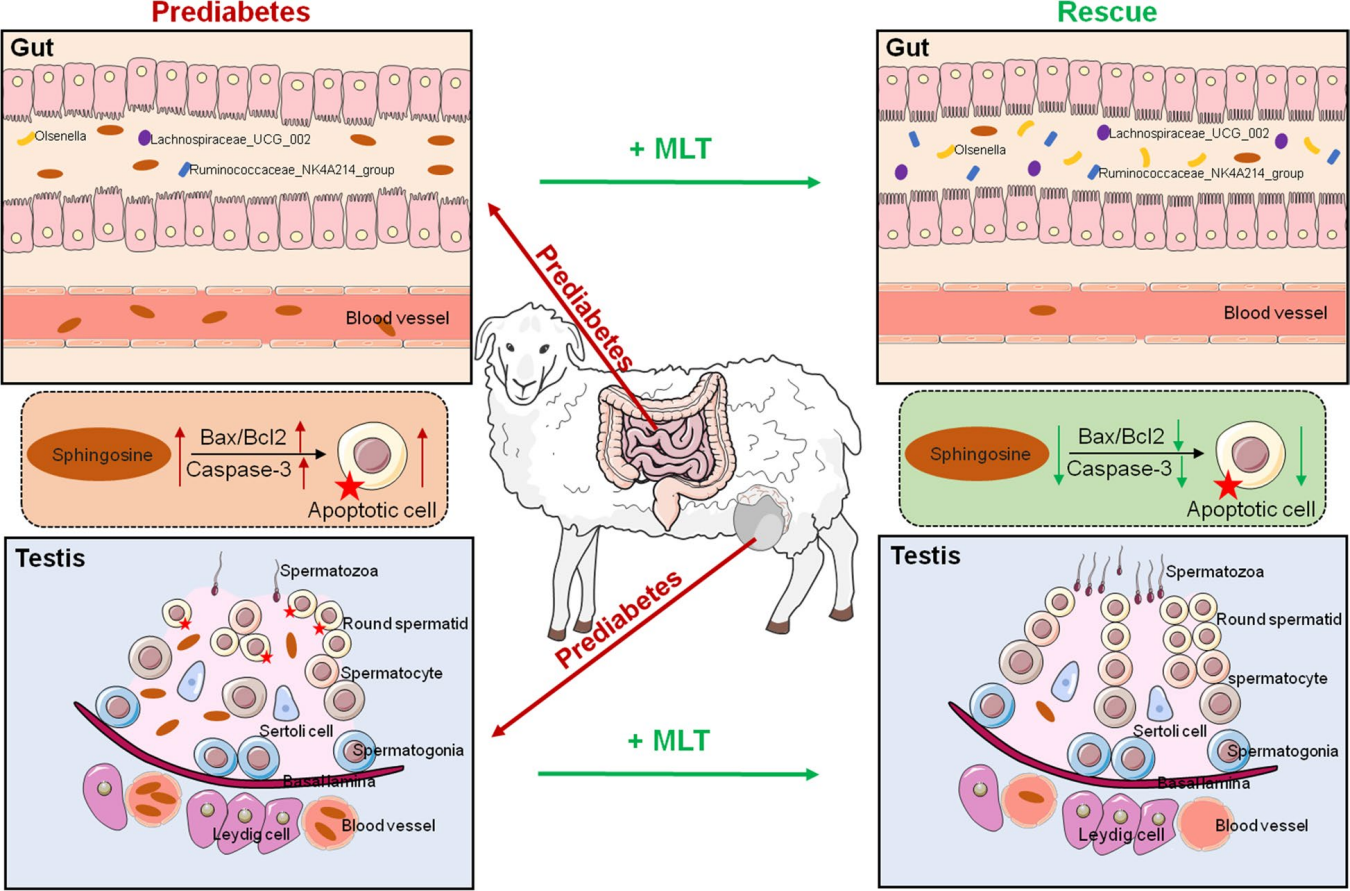
Diagram illustrating the proposed sphingosine accumulation caused by gut microbiota dysbiosis leads to damage to the spermatogenic process. The accumulation of sphingosine, mainly owing to the dramatically decreased abundance of *Olsenella*, *Lachnospiraceae_UCG_002* and *Ruminococcaceae_NK4A214_group* in the prediabetic sheep model. The accumulation of sphingosine was transferable to the testis through the blood circulation, which increased the level of apoptosis of testicular tissues, resulting in the arrest of S2 Round Std. Meanwhile, adding melatonin restored the status of gut microbiota dysbiosis and sphingosine accumulation in the prediabetic sheep model, as well as improving abnormal spermatogenesis

All the above results suggest that microbiota dysbiosis caused by prediabetes leads to the accumulation of sphingosine, which increases the level of apoptosis in testicular tissues. Furthermore, an arrest in the transformation of S2 to S3 stage round spermatids results in abnormal spermatogenesis, while the supplementation of melatonin was able to protect against this injury (Fig. 7).

## Discussion

Greater attention is being paid to the occurrence of impaired spermatogenesis in diabetic patients, but the influence of undiagnosed prediabetes on spermatogenesis has been less studied. Current studies have shown that spermatogenesis and sperm quality will be affected in both type 1 and type 2 DM patients [4–9]. Abnormal glucose metabolism leads to impaired repair of sperm DNA damage and decreased sperm motility. In this study, we established a prediabetic sheep model, whose fasting insulin and glucose levels were significantly increased, and the insulin resistance index was also significantly increased. We found that there was an obvious inhibition of spermatogenesis in the prediabetic testis, and the quality and quantity of spermatogenic cells was significantly decreased. Other studies also reported a similar trend; Boeri et al. found that there were about 15.4% undiagnosed prediabetic patients in investigative 744 infertile men; what is more, prediabetic patients were proved to have higher fragmentation index of sperm DNA, as well as higher level of idiopathic non-obstructive azoospermia compared to those without prediabetes [13]. In this study, we performed single cell transcriptome analysis to establish the gene expression profiles of testicular cells; we found that there was an obvious arrest in round spermatid development in the prediabetes group, which was consistent with testicular test results.

Metabolic disorders in diabetic patients lead to a variety of complications, including hyperglycemia and glycosuria caused by abnormal glucose metabolism, hyperlipidemia, and ketoacidosis as a result of abnormal lipid metabolism [31]. In this study, we performed metabolomics analysis on intestinal digesta of the three groups and found that differential metabolites were mainly enriched on the apoptosis and sphingolipid metabolism pathways. Sphingosine is a metabolite of sphingomyelin, which plays a role in promoting cell apoptosis. Thus, we examined the levels of sphingomyelin and sphingosine in the serum and testis of the three groups and found that they were increased in the prediabetes. Furthermore, sphingosine accumulation increased the apoptotic levels of testicular tissues. Gut microbiota plays an important role in substance metabolism. Our data revealed that gut microbiota dysbiosis in the prediabetic model contributed to the abnormal metabolism of sphingosine. These results revealed that gut microbiota dysbiosis induced sphingosine accumulation in the prediabetes group that impaired spermatogenesis, which was also verified in mice by FMT.

Melatonin is one of the hormones secreted by the pineal gland of the brain; it has a regulatory effect on the immune system, nervous system, and cardiovascular system [32]. Studies have confirmed that melatonin can effectively improve fasting blood glucose and oxidative stress levels in DM patients [21, 22]. In this study, we administered melatonin to create a rescue group and found that melatonin significantly protected against increasing levels of blood glucose and insulin resistance in prediabetic sheep. In terms of testicular spermatogenesis, melatonin effectively restored this process and increased the proportion of MVH-positive cells in the seminiferous tubules. Compared with the prediabetes group, the pseudotime distribution of round spermatids in the rescue group were similar to those in the control group, and the arrest trend from S2 Round Std to S3 Round Std disappeared. Both the metabolism of sphingosine and the composition of gut microbiota in the rescue group were similar to those of the control group, showing a significant protective effect. This trend was also verified in mice by FMT. Compared with prediabetes-FMT mice, spermatogenesis and apoptosis of Sertoli cells were significantly restored in rescue-FMT mice.

## Conclusions

Our study confirmed that gut microbiota dysbiosis in a prediabetic model led to abnormal metabolism of sphingosine, and sphingosine accumulation in turn caused damage to spermatogenesis and increased the level of apoptosis in testicular tissues; meanwhile, adding melatonin effectively alleviated these adverse lesions. Our study explored and established a gut-testis axis model in the prediabetic condition, which suffered from disordered spermatogenesis, thus demonstrating the relationship between gut microbiota and substance metabolism, and their effects on spermatogenesis. In addition, we attempted to add a therapeutic drug to rescue spermatogenesis through remodeling gut microbiota and metabolism; this will be an important finding for clinical research and treatment of prediabetic infertility.

## Methods

### Establishment of prediabetic sheep model

All animal procedures were approved and conducted in accordance with the Inner Mongolia University Animal Care and Use Committee. Mongolia male sheep were allowed to eat and drink freely for at least 1 week to adapt to the environment. Sheep were randomly divided into three groups, there were five sheep in each of the three groups. The control group sheep were fed with a standard chow diet, the prediabetes group sheep were fed with a high-concentrate diet, and the rescue group sheep were fed with a high-concentrate diet adding melatonin (10 mg/kg/day) (Supplementary Table 1). We give melatonin to sheep by regular and quantitative watering. Approximately 50 testicular samples were prepared from each sheep for qPCR, WB, HE, IHC, IF, TUNEL, and ELISA assay.

### Single cell transcriptome analysis by 10 × genomics

Single cell libraries were prepared using the 10 × Genomics Chromium Single Cell 3′Library & Gel Bead Kit v2 (10 × Genomics Inc., USA) following the manufacturer’s instructions. Briefly, we obtained testicular cells from freshly isolated testicular samples. Testes of three group of sheep were collected and washed for three times with PBS. Then, the testes were cut into pieces and digested in collagenase type IV (1 mg/ml) at 37 °C for 15 min. And 15 min later, DMEM with 10% FBS was used to stop the digestion process. After being washed for two times, the samples were mechanically separated into a single cell suspension. The cell suspension was filtered with a cell strainer (40 μm). Then, the cells were washed for three times in PBS solution supplemented with 0.04% BSA. After, Trypan blue staining and cell count were done to confirm the suitable viability (> 80%) and concentration (1000 cells/μl). The testicular cells of three groups were underwent treatment to form the Gel-Bead in Emulsions system through Chromium 10X Single Cell System, in which the cells were barcoded. We obtained more than 8000 cells to prepare the single cell library from mixed testicular samples, which were from three sheep of each group. Construction of cDNA library was performed following the instruction using the 10 × Genomics Chromium Single Cell 3′Library & Gel Bead Kit v2. We used Illumina NovaSeq 6000 to carry out the sequencing process with pair end 150 bp (PE150) reads. For the data analysis, we used Seurat software (v3.1.5) to cluster cell type and value gene expression profiles of different cell type and used Monocle 2 (v2.16.0) to determine the single-cell pseudo-time trajectory.

### Examination of metabolomic profiles of intestinal digesta samples

The intestine digesta samples of three groups were collected and mixed with extract solution. Then, the mixture were homogenized for 4 min at 35 Hz, followed by sonicated for 5 min in ice-water bath. The samples were incubated for 1 h at − 40 °C and were centrifuged at 12,000 rpm for 15 min at 4 °C. The supernatant was collected and dried in a vacuum concentrator at 37 °C. After sonication on ice for 10 min reconstituted adding 50% acetonitrile, the samples were centrifuged at 13,000 rpm for 15 min at 4 °C. The supernatants were collected and examined by UHPLC-QTOF-MS to measure the metabolomics profiles. We used 1290 Infinity series UHPLC System for Liquid chromatographic separation on a UPLC BEH Amide column, and 25 mM ammonium acetate and 25 mM ammonia hydroxide constructed the mobile phase. We used TripleTOF 6600 mass spectrometry system for LC/MS experiment.

### Real-time quantitative PCR (qPCR) analyses

Total RNA was extracted using an EasyPure RNA Kit (TRANS, ER101-01), and cDNAs were synthesized using a Reverse Transcription kit (QIAGEN, 205,311). qPCR was performed with the TB Green Premix Ex TaqII (TAKARA, RR820A). The primer for *Sphk1* (forward: 5′-ACC TCC TGA CCA ACT GCA CGC A-3′ and reverse: 5′-TCG CTC TCC AGA TCC ACA TCG G-3′), *Sgpl-1* (forward: 5′-TGA GGA TGG CTT GTT CCC TAT TCA-3′ and reverse: 5′-GGG GGC TAC GAT TTC TGG AGT TTT-3′), *β-actin* (forward: 5′-TCC TGC GGC ATT CAC GAA ACT A-3′ and reverse: 5′-TCC TGC TTG CTG ATC CAC ATC TG-3′), *Bax* (forward: 5′- CAG GAT GCG TCC ACC AAG AA-3′ and reverse: 5′- GCA AAG TAG AAG AGG GCA ACC AC-3′), *Bcl-2* (forward: 5′- TGG TGG AGG AAC TCT TCA GGG-3′ and reverse: 5′- AGG AGA AAT CAA ACA GAG GTC GC-3′), *Caspase3* (forward: 5′- GTC TGA CTG GAA AGC CGA AAC TC-3′ and reverse: 5′- AGG GAC TGG ATG AAC CAC GAC-3′), and *Gapdh* (forward: 5′- GCT ACA CTG AGG ACC AGG TTG TCT-3′ and reverse: 5′- GAG GTC CAC CAC CCT GTT GC-3′) were used. The relative expression of *Sphk1* and *Sgpl-1* gene was rectified by expression of *β-actin*, and the relative expression of *Bax*, *Bcl-2*, and *Caspase3* gene was rectified by expression of *Gapdh*.

### TUNEL assay

A DeadEnd Fluorometric TUNEL System (Promega, G3250) was used in TUNEL assay. According to the instructions, tissue sections were stained with sufficient rTdT incubation buffer at 37 °C for 60 min in a humid atmosphere, protecting from direct light exposure. The tissues were covered with plastic coverslips to ensure even distribution of the reagent. Nuclei were counterstained with DAPI. The tissue sections were randomly visualized on a fluorescence microscope at × 40 magnification.

### ELISA assay

For serum and testicular sphingosine measurements, serum samples and testes homogenate were processed for sphingosine concentration assays by an ELISA kit (BYabscience, BY-99863).

### Western blot analysis

The three group sheep testes were collected and extracted in cold RIPA buffer on ice, which supplemented with phenylmethylsulfonyl fluoride and protease inhibitor to obtain protein extracts. The protein extracts were resolved by SDS-PAGE and then transferred onto an Immobilon-PSQ Transfer Membrane (Millipore, IPVH00010, Germany) and probed with the primary antibodies at 4 °C overnight. Antibodies were diluted as follows: MVH (abcam, AB13840, 1:1000), β-ACTIN (Sigma, A1978, 1:1000). Then, the membranes were washed in TBST, following an incubation with secondary antibody in TBST, at 37 °C for 2 h. Then, was used for the exposure.

### Morphology and immunofluorescence/immunohistochemical

The three group sheep testes were collected, and fixed in 4% paraformaldehyde at 4 °C overnight, and then dehydrated and embedded in paraffin. The testis or epididymis samples were sectioned into 5-μm sections. Before used, the sections were deparaffinized by dimethylbenzene and ethanol; for morphology assay, the sections were prepared and stained with hematoxylin and eosin. For immunofluorescence/immunohistochemical, the sections were transferred into sodium citrate buffer at 96 °C for 10 min. Samples were then blocked with 5% BSA in TBST for an hour and probed with the primary antibodies MVH (abcam, AB13840, 1:200) at 4 °C overnight. After washing with TBST three times, the sections were incubated with secondary antibodies labeled by fluorescent group or horseradish peroxidase (tissues and primary antibodies were treated with 1% hydrogen peroxide) for an hour at 37 °C. Counterstaining was treated with DAPI for 5 min/hematoxylin for 2 min. Sections were examined under a Nikon fluorescence microscope (A1, Japan). Within the group, five testicular cross-sections which were at least 50 sections apart from each animal were examined.

### 16S rDNA sequencing of intestinal digesta bacterial

We used a QIAGEN kit to extracted the genomic DNA of intestine microbiota samples. After quantification by Qubit 2.0 fluorometer and generating amplicon of 16S rDNA, we recovered DNA libraries by GeneJET Gel Extraction Kit and quantified using a Qubit 2.0 fluorometer. The obtained DNA libraries were sequenced using the 250 bp paired-end strategy on the Illumina platform. To get the raw tags, paired-end reads were merged by FLASH (V1.2.7). Then, raw tags were filtered and clustered by FASTX-Toolkit. We identified possible chimeras by employing UCHIME. The denoised sequences were clustered using USEARCH (version 10.0) and tags with similarity ≥ 97% were regarded as an operational taxonomic units (OTUs) using MOTHUR pipeline. Taxonomy was assigned to all OTUs by searching against the Silva databases using the uclust within QIIME1.8.0. The Principal coordinate analysis (PCoA), taxonomy, and heatmap of differential abundance were calculated and visualized using R software.

### Fecal microbiota transplantation

Microbiota in small intestine were collected from three group of sheep. Specific steps are as follows: 1 g fecal was mixed with 1 ml sterile glycerin (20%), then the mixture were underwent homogenized and frozen. The fecal sample was diluted to a working concentration of 0.05 g/mL with sterile normal saline and filtered with a 70 μm cellular filter. It is necessary that fresh graft materials were prepared within 10 min before intragastric administration to keep the steady of bacterial composition. More than 10 male ICR mice aged 3 weeks were used in each group of this study. Before the fecal transplantation, receptor mice were treated with antibiotics [vancomycin (0.5 g/liter), neomycin sulfate (1 g/liter), metronidazole (1 g/liter), and ampicillin (1 g/liter)] for 2 weeks. Oral gavage with fresh graft material was given daily for 8 weeks.

### Statistics

We used GraphPad Prism for statistical analysis. The data of biological assay are represented as mean ± SEM. *T*-test was used to assess the difference between two groups (normal distribution) and one-way analysis of variance (ANOVA) for multiple comparison tests. All results were considered statistically significant at *P* < 0.05. Differential metabolites were defined as those with variable importance in the projection (VIP) > 1.0 and adjusted *P* < 0.05.

## Supplementary Information

**Additional file 1.**

## Data Availability

All data relevant to the study are included in the article or uploaded as supplementary information. The 10 × Genomics single cell RNA sequencing data of sheep testis have been deposited in Genome Sequence Archive (GSA) with the accession number CRA005278 [33]. The 16S rDNA sequencing data of intestinal digesta have been deposited in GSA with the accession number CRA005272 [34]. The metabolomic profiles of intestinal digesta data have been deposited in the OMIX, China National Center for Bioinformation/Beijing Institute of Genomics, Chinese Academy of Sciences [35].

## Abbreviations

FMT: Fecal microbiota transplantation
DM: Diabetes mellitus
FSH: Follicle-stimulating hormone
SPH: Sphingosine
S1P: Sphingosine-1-phosphate
Round Std: Round spermatid
HDL: High-density lipoprotein
LDL: Low density lipoprotein
LIPC: Hepatic lipase
CHOL2: Total cholesterol
MVH: Mouse vasa homolog
SPG: Spermatogonia
SPC: Spermatocyte
UHPLC/Q-TOF MS: Ultra-high performance liquid chromatography-quadrupole time-of-flight mass spectrometry
PLS-DA: Partial least squares discriminant analysis
OTUs: Operational taxonomic units
PCoA: Principal coordinate analysis
ANOVA: One-way analysis of variance
