# Supplementary material for: Elevated testicular apoptosis is associated with elevated sphingosine driven by gut microbiota in prediabetic sheep
Source: BMC Biol. 2022 May 24;20:121. doi: 10.1186/s12915-022-01326-y (PMC9128135; doi:10.1186/s12915-022-01326-y)

Supporting Information

Elevated testicular apoptosis is associated with elevated sphingosine driven by gut microbiota in prediabetic sheep

Yuanchao Sun[1,2,#], Peng Sun[1,3,#], Yanting Hu[1,#], Liying Shan[1,#], Qi Geng[1], Yutian Gong[1], Haitao Fan[1], Teng Zhang[1,], Yang Zhou[1,]**

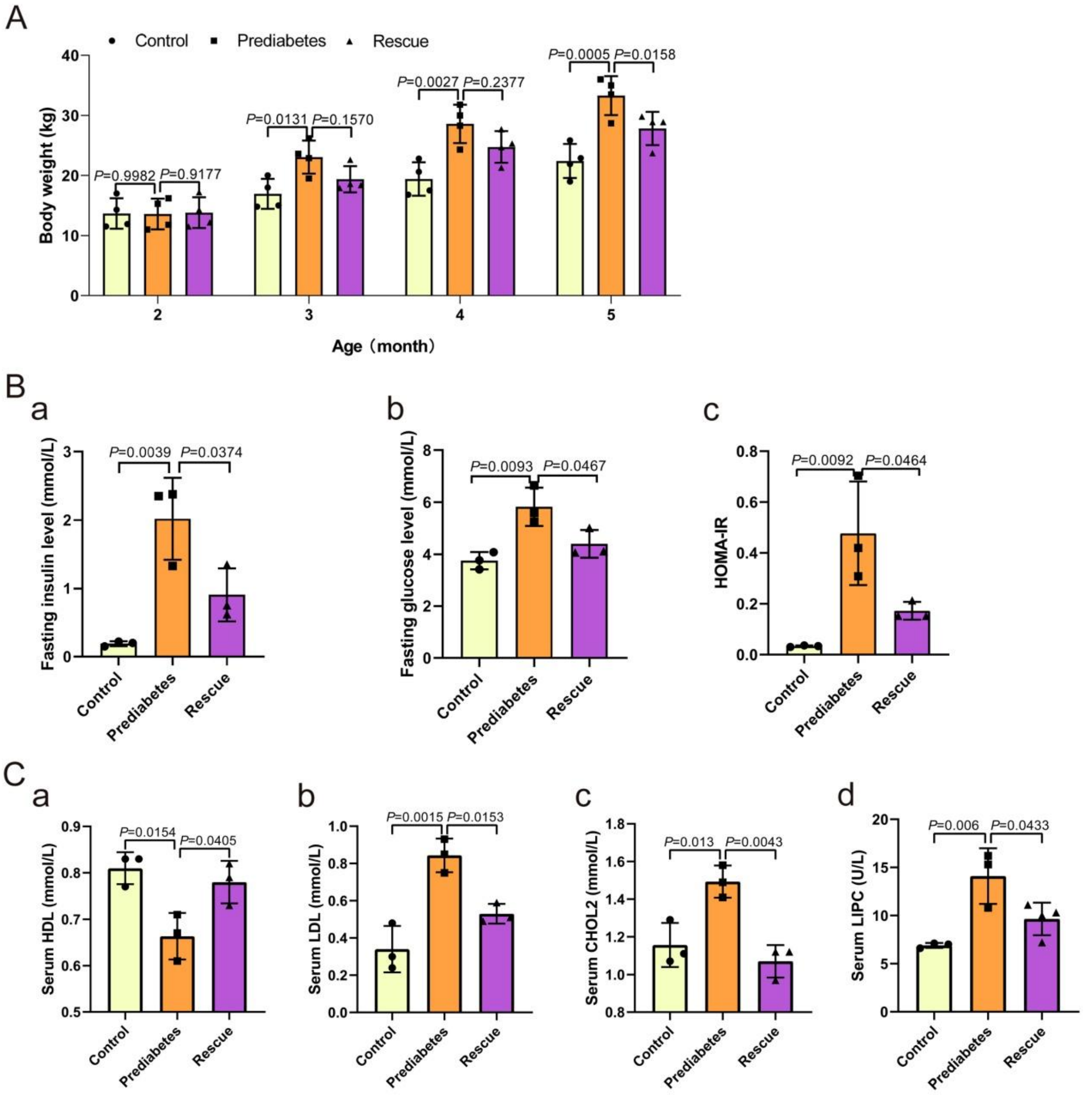

Figure S1. Physiological indicator alteration in the prediabetic sheep model. **A** Body weights for the three groups, with age (*n*=4). **B** (a) Fasting insulin levels (*n*=3), (b) fasting glucose levels (*n*=3), and (c) HOMA-IR insulin resistance index (*n*=3) in the control, prediabetes, and rescue groups. **C** Levels of (a) HDL (*n*=3), (b) LDL (*n*=3), (c) CHOL2 (*n*=3), and (d) LIPC (*n*=3) in sera of the three groups.

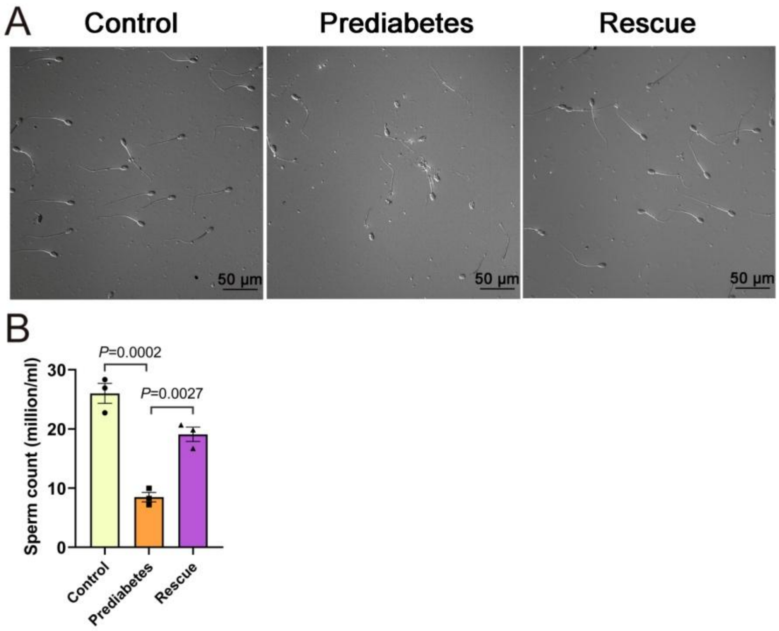

Figure S2. The sperm parameters. **A** The sperm smear of the spermatozoa morphology. Scale bar = 50 μm. **B** Statistical analysis of sperm counts from three groups (n=3).

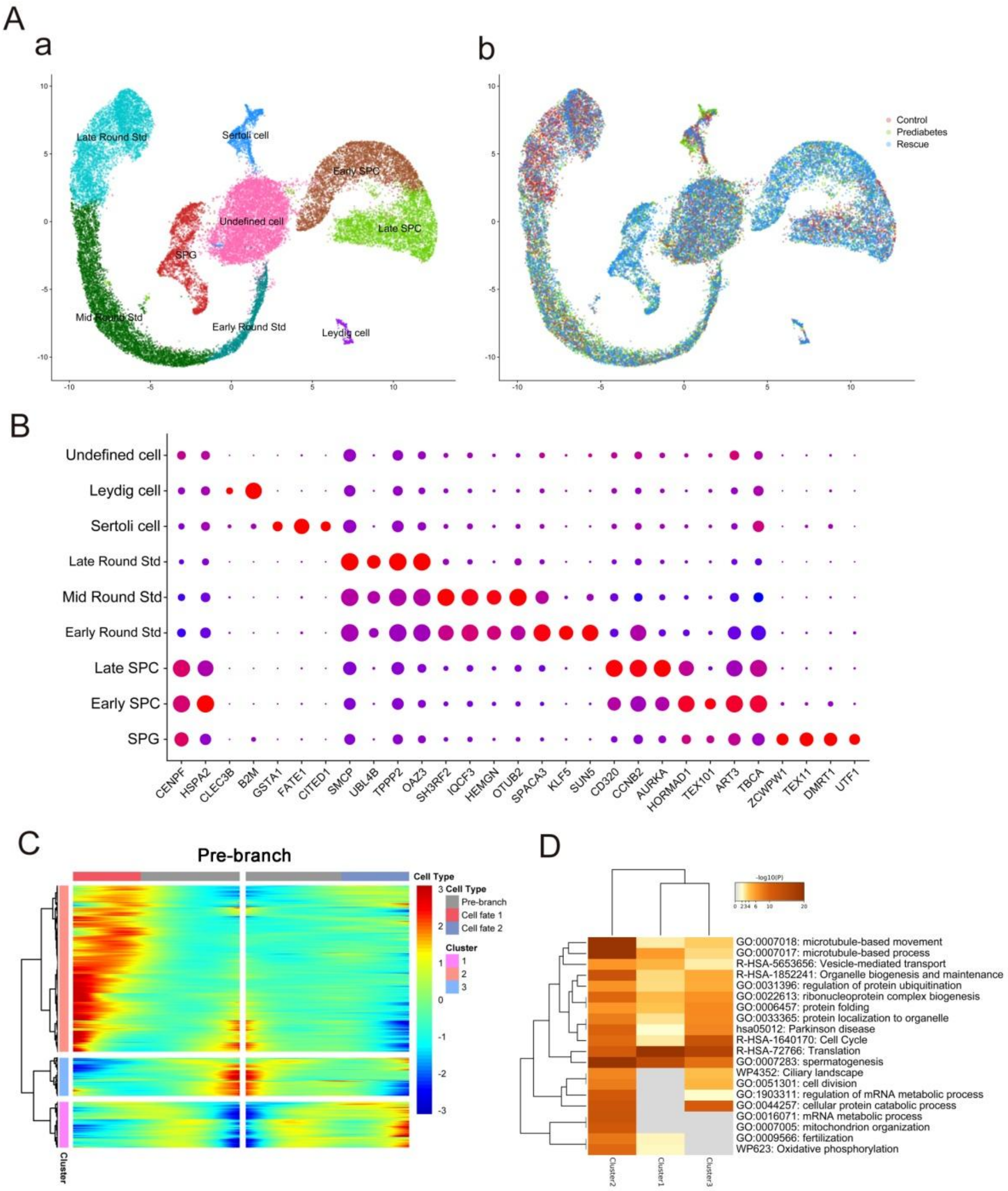

Figure S3. Cluster identification of sheep testicular cells. **A** (a) UMAP plot of testicular cells as defined by scRNA-seq analysis. (b) UMAP plot of three group testicular cells. **B** Dot plot of general special marker genes expressed in each cluster. **C** Heatmap represents significant DEGs between each cluster of Round Std arrest at branch point 1. **D** Gene Ontology (GO) terms of DEGs in three gene clusters.

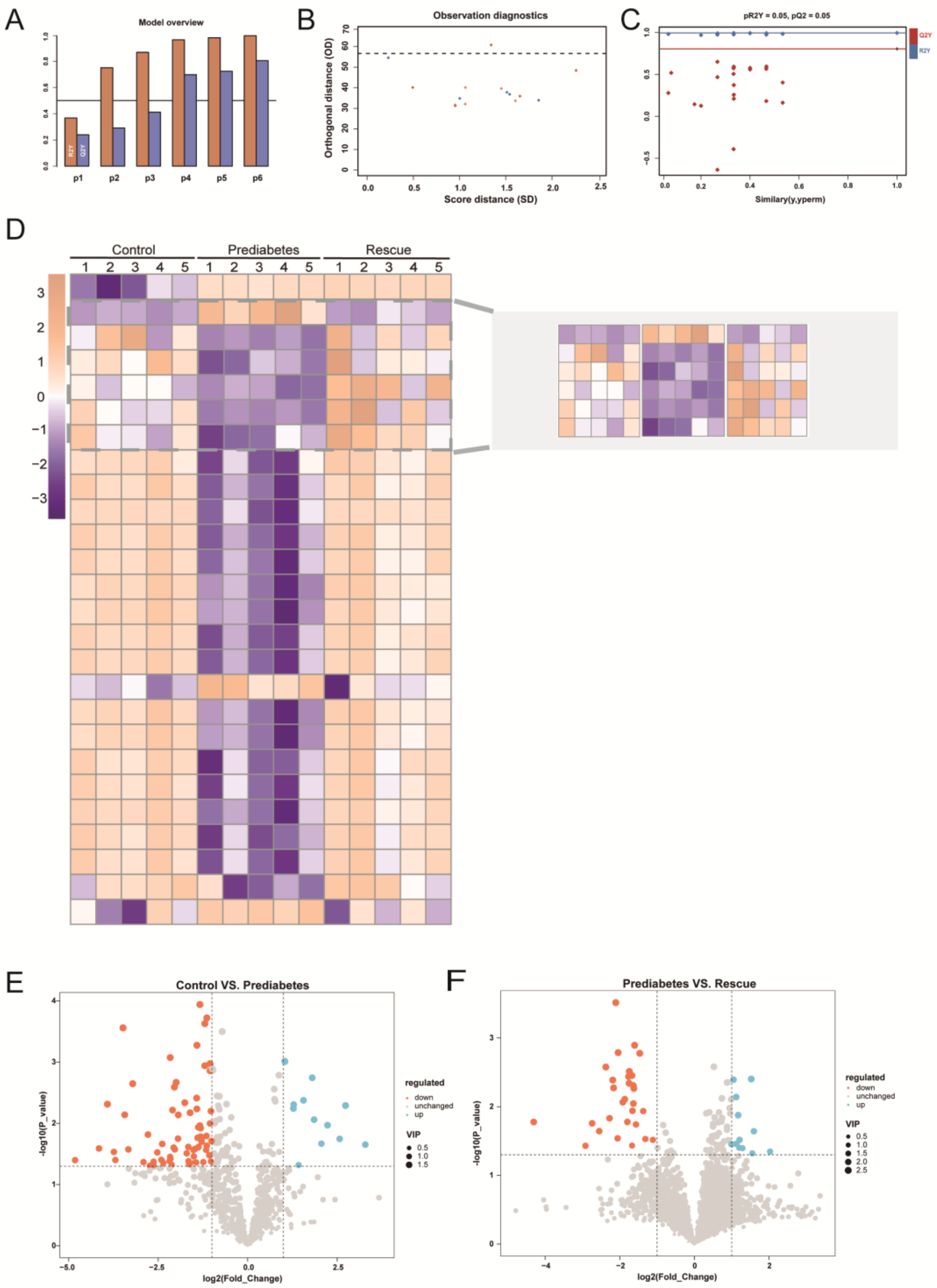

Figure S4. Metabolomics profiling. **A-C** PLS-DA validation diagram. **D** Heatmaps of general differential metabolites. **E-F** Volcano plots of general differential metabolites in control vs. prediabetes groups and prediabetes vs. rescue groups (*n*=5).

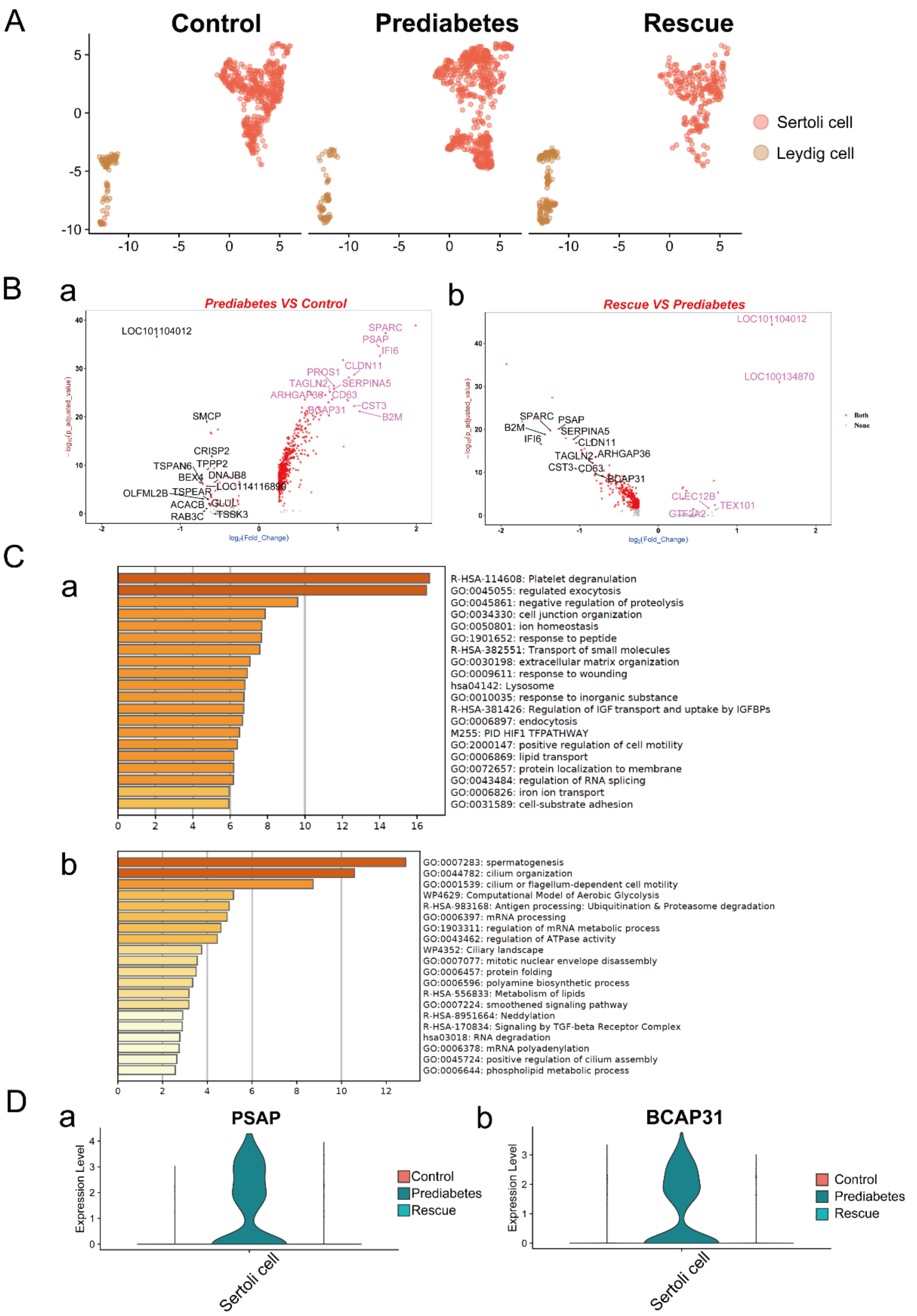

Figure S5. Transcript profile of Sertoli cells in the three groups. **A** UMAP plot of Sertoli cells in the three groups. **B** Volcano plot demonstrating DEGs in the prediabetes group vs. control, and rescue group vs. prediabetes group. **C** GO terms of up-regulated (a) and down-regulated (b) genes in the prediabetes vs. control groups. **D** Vlnplots of (a) *Psap* and (b) *Bcap31* in the three groups.

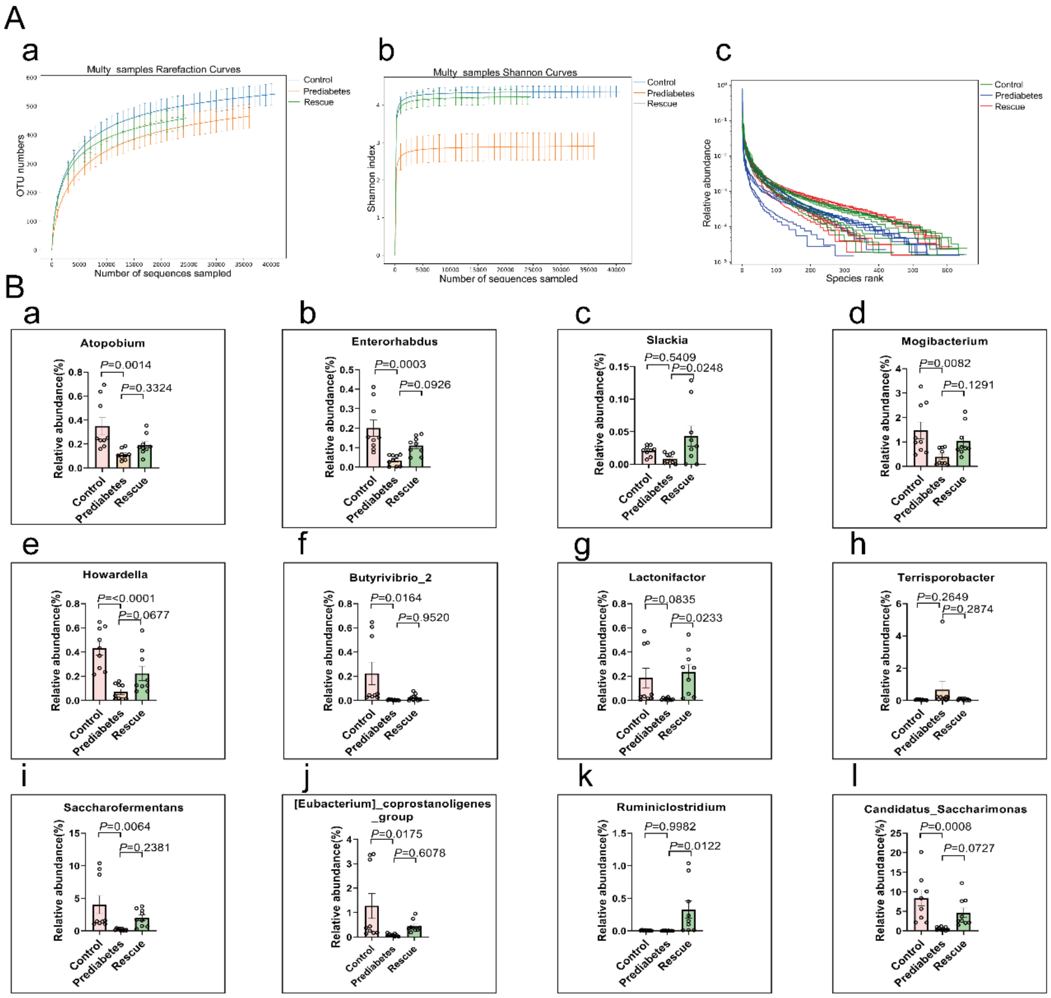

Figure S6. α-diversity of gut microbiota and the abundance of genus-level gut microbiota in the three groups. **A** (a) OTU rank curves, (b) Shannon curves, and (c) rarefaction curves of gut microbiota for samples of the three groups. **B** Comparison of the relative abundance of genus level bacteria (a) *Atopobium*, (b) *Enterorhabdus*, (c) *Slackia*, (d) *Mogibacterium*, (e) *Howardella*, (f) *Butyrivibrio_2*, (g) *Lactonifactor*, (h) *Terrisprobobacter*, (i) *Saccharofermentans*, (j) *[Eubacterium]_coprostanoligenes_group*, (k) *Ruminiclostridium*, and (l) *Candidatus_Saccharimonas* in the three groups (*n*=9).

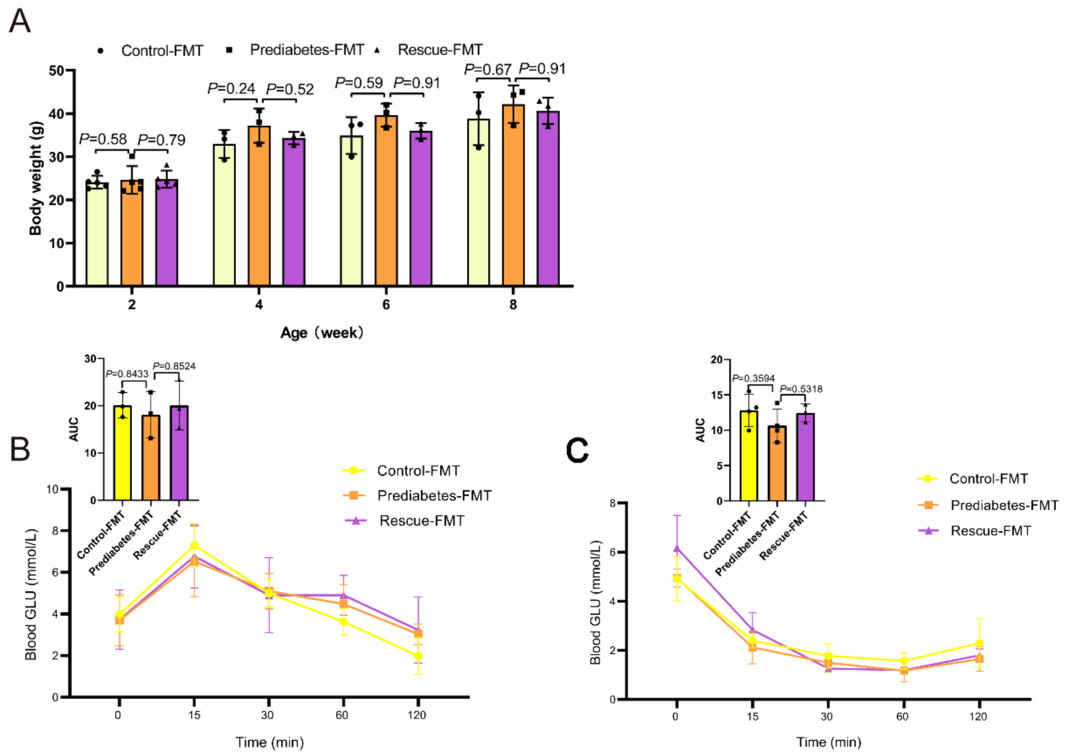

Figure S7. Physiological indicators in FMT mice. **A** Body weight in the three FMT groups with age (*n*=3). **B** Glucose tolerance test (*n*=3). **C** Insulin tolerance test (*n*=3).

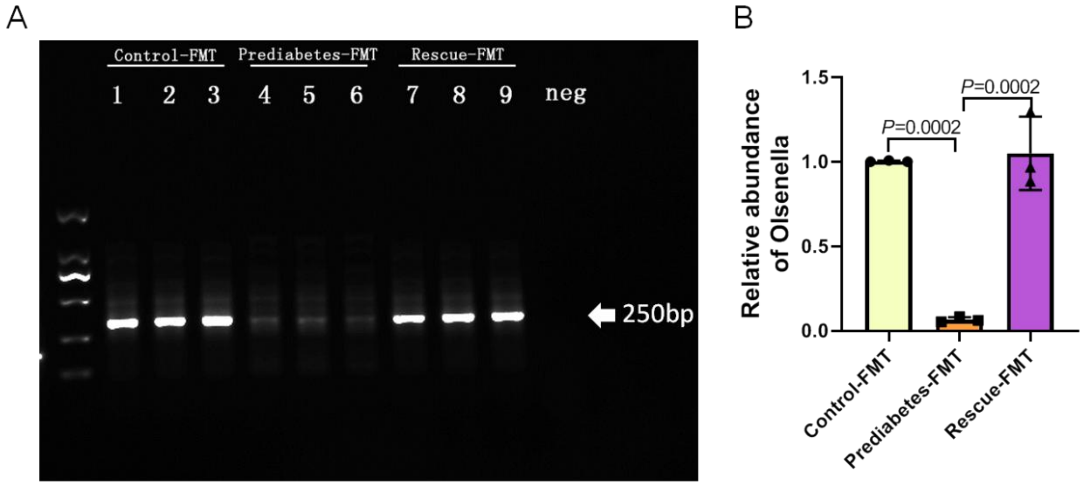

Figure S8. Comparison of the relative abundance of *Olsenella*. **A** The PCR analysis of *Olsenella* abundance in Control-FMT, Prediabetes-FMT and Rescue-FMT groups (n=3). **B** The Q-PCR analysis of *Olsenella* abundance in the three FMT groups (n=3).

Supplementary table 1 The percentage of feedstuff composition

Composition / Group	Control	Prediabetes	Rescue
**Corn**	8.29%	25.31%	25.31%
**Soybean meal**	8.47%	25.85%	25.85%
**Premix**	0.88%	0.88%	0.88%
**Alfalfa**	10.29%	5.77%	5.77%
**Ensilaged straw**	72.07%	42.19%	42.19%

Supplementary material

The original full-length blots from which the cropped images are derived

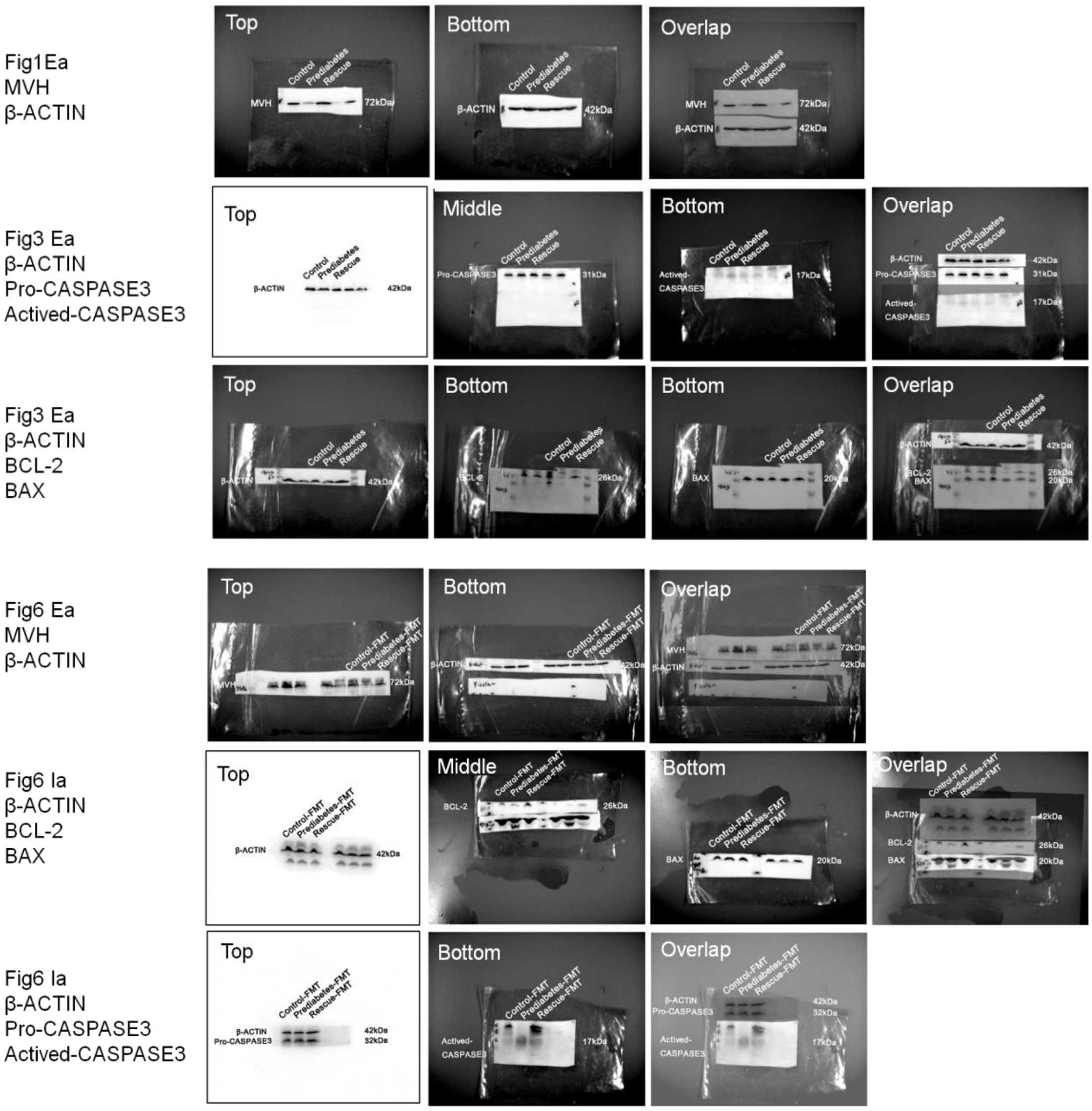

Supplement: Supplementary file 1 — Additional file 1. [file 12915_2022_1326_MOESM1_ESM.pdf]
